# Compatible solute, transporter protein, transcription factor, and hormone-related gene expression provides an indicator of drought stress in *Paulownia fortunei*

**DOI:** 10.1007/s10142-014-0373-4

**Published:** 2014-05-07

**Authors:** Yanpeng Dong, Guoqiang Fan, Zhenli Zhao, Minjie Deng

**Affiliations:** 1 Institute of Paulownia, Henan Agricultural University, 95 Wenhua Road, Jinshui Area, Zhengzhou, Henan People’s Republic of China 450002; 2 College of Forestry, Henan Agricultural University, 95 Wenhua Road, Jinshui Area, Zhengzhou, Henan People’s Republic of China 450002

**Keywords:** Drought, *Paulownia fortunei*, Diploid, Autotetraploid, Transcriptome

## Abstract

Drought is one of the most devastating effects of global climate change. Leaves contribute significantly to the management of water deficit and plant adaptation to drought stress. In this study, we compared the transcriptomes of leaves of two genotypes of *Paulownia fortunei* with different drought tolerances. Solexa sequencing and qRT-PCR were used for gene expression analysis and validation. Variations in leaf growth were found between drought-treated and well-watered samples in both genotypes. Drought-treated samples from diploid and autotetraploid *P. fortunei* cultivars showed inward leaf rolling and smaller blade size compared with the well-watered ones. High throughput transcriptome sequencing generated 266,700,100 high-quality reads representing 110,586 unigenes from the leaves. The drought-treated samples responded to water deficiency by inducing various genes and pathways, such as photosynthesis, carbon fixation in photosynthetic organisms, stress response, plant hormone signal transduction, and flavonoid pathways. Regulatory genes, such as WRKY, and transcription factors, such as NAC, known for leaf development under drought stress, were highly expressed in the drought-treated samples, and so were the genes related to compatible solutes (sugars, sugar alcohols, amino sugars, amino acids, or betaine), hormones, and various transporters. This study illustrates changes in the expression pattern of genes induced in response to drought stress and provides a comprehensive and specific set of drought-responsive genes in *P. fortunei*.

**Electronic supplementary material:**

The online version of this article (doi:10.1007/s10142-014-0373-4) contains supplementary material, which is available to authorized users.

## Introduction

Plants are constantly exposed to water limitation, which negatively affects plant development and growth; therefore, understanding the mechanism of plants’ water use will provide molecular basis for the development of plant varieties that are better adapted to drought stress. Leaf plays an important part in adaptability of plants to water stress (Terzi et al. 2009; Guo et al. 2004; Luo et al. 2010). Leaves of plants in water-deprived environments show reduced transpiration, which results from a variety of sources, including closure of stomata pores, leaf rolling, and withering. In such cases, one of the major responses of plants to water deprivation at the molecular level is the induction of drought-responsive and drought-tolerant genes (Ashoub et al. 2013).

To gain a comprehensive perspective on the molecular mechanisms and related genes underlying drought tolerance, one of the most effective methods is to study the genome-wide transcriptional patterns under drought stress. In recent studies, gene expressions in leaves undergoing water deprivation have been comprehensively studied in the model plant *Arabidopsis*, in crops such as rice and maize, and in other herbaceous plants (Wilkins et al. 2010; Wang et al. 2011; Zheng et al. 2010; Pratt et al. 2005). By contrast, there have been few studies on woody plant species. In the recent years, many works referred to the transcriptomic responses to drought stress of *Populus* species. Some of those studies have focused on the identification of genes induced by stress (Caruso et al. 2008; Bae et al. 2010), while others examined the expression pattern and function of specific genes (Bae et al. 2009, 2011; Chen et al. 2011). Exhaustive transcriptome studies using massive sequencing have also been published (Hamanishi and Campbell 2011). Although not in the same depth as in poplar, transcriptomic response to drought has also been studied in other tree species, such as *Jatropha curcas*, *Quercus* spp., *Picea*, and *Pinus* (Sapeta et al. 2013; Gimeno et al. 2009; Costa et al. 2010; Gailing et al. 2009).

The identified drought-induced genes from these transcriptomic analyses can usually be classified into three large groups based on their biological roles: (1) genes encoding proteins that function in abiotic stress tolerance, (2) genes encoding regulatory proteins, and (3) genes involved in growth and development (Upadhyaya et al. 2013; Tuteja et al. 2011; Aranda et al. 2012). The first group comprises genes encoding chaperones, sugar and proline transporters, compatible solutes (e.g., glycine betaine and proline), osmotin, late embryogenesis abundant proteins, mRNA-binding proteins, water channel proteins, ion channels, key enzymes for osmolyte biosynthesis, detoxification enzymes (e.g., superoxide dismutase (SOD), glutathione peroxidases, peroxidases, catalases, and peroxiredoxins) and various proteases. The regulatory protein genes in the second group include transcription factors (such as AP2/EREBP, WRKY, MYB, NAC, DREB/ CBF, zinc finger, bHLH, and bZIP/AREB/ABF as proteins), which are involved in the regulation of downstream genes, protein kinases, protein phosphatases, enzymes involved in phospholipid metabolism, and the components of calcium-coupled phosphoprotein cascades and other proteins regulating RNA metabolism and stability (such as DEAD box RNA helicases), translation (ribosomal proteins), and protein degradation (proteases/protease inhibitors, ubiquitin ligase). The third group includes genes encoding cell components and various hormones such as auxin and cytokinin, which serve as regulators of plant growth and development.

*Paulownia fortunei* is a fast-growing hardwood species grown in dry, marginal areas. It is a native species to Asia with a history of over 2,000 years and has also been introduced into Europe, America, and Australia (Krikorian 1988; Lyons 1993). In most planting areas, *Paulownia* is produced in regions where water is limited (Lyons 1993). Such environmental conditions suggest a high potential for genetic adaptation to water stress in this species. Understanding the molecular mechanisms of the response and adaptation of *Paulownia* to drought stress is of prime importance.

Natural *P. fortunei* is a diploid (2*n* = 40) plant. Polyploid plants display many improved properties (Adams et al. 2003), and to investigate whether this also applies to *Paulownia*, a system of in vitro plantlet regeneration from the leaves of tetraploids (4*n* = 80) of *P. fortunei* was established (Yang et al. 2007b). The tetraploid has improved characteristics compared with the diploid, including improved drought tolerance. A detailed physiological study on tetraploids and diploid has also been conducted (Fan et al. 2007).

In the current study, we report the expression profiling of the leaves of two genotypes of *P. fortunei*, diploid, and autotetraploid under well-watered and drought-treated conditions, respectively, using Illumina’s Solexa sequencing. We identified various stress-responsive genes and inherent biochemical pathways that reflect the probable mechanisms of the adaptive advantage of the drought-treated samples. In drought-prone areas in Asia, PF4W75 is potentially a better selection because of their ability to withstand drought.

## Materials and methods

### Plants

All plant materials used in the study were obtained from the Institute of Paulownia, Henan Agricultural University, Zhengzhou, Henan Province, China. The tissue culture seedlings of diploid and tetraploid *P. fortunei* were cultured for 30 days before being clipped from the roots. Samples were transferred into nutrition blocks containing normal garden soil for 30 days. Samples with the same growth consistency were then transferred individually into nutrition pots of 30 cm in diameter with trays underneath. Each pot was filled with the same amount of ordinary garden soil for each plant. After 50 days, the tissue culture seedlings with the same growth consistency were subjected to drought treatment in a water control experiment according to the method of Zhang et al. (2013). Diploid and tetraploid *Paulownia* with 25 and 75 % relative soil water contents were named PF2W25 and PF2W75, PF4W25 and PF4W75, respectively.

### Physiological responses of diploid and tetraploid *Paulownia* to drought stress

After 3, 6, 9, and 12 days (wilting state), respectively, the second pairs of leaves from the growing apex of the young sprout of the plants were picked from the drought-treated samples. The corresponding diploid leaf samples were renamed PF2W25-3D, PF2W25-6D, PF2W25-9D, and PF2W25-12D, respectively, while the corresponding tetraploid ones were renamed PF4W25-3D, PF4W25-6D, PF4W25-9D, and PF4W25-12D, respectively. The well-watered samples were picked after 12 days only. At least three parallel samples were prepared for each condition. The relative water content was measured by Drying Weighing Method (Liu and Liu 2011). The chlorophyll content was measured by Ethanol Method. The superoxide dismutase (SOD) activity was measured by Pyrogallol Autoxidation Method. The soluble protein content was measured by Coomassie Brilliant Blue Method. The proline content was measured by Potentiometric Titration Method. The methods adopted above were according to the methods by Lu and Li (2012).

### Construction of *Paulownia* cDNA libraries

For each condition, approximately 8 mg of leaves were homogenized in liquid nitrogen with a pestle. Total RNA was extracted from the cells using the TRIzol reagent (Invitrogen, Carlsbad, CA, USA), followed by RNA purification using an RNeasy MiniElute Cleanup Kit (Qiagen, Dusseldorf, Germany), according to the manufacturer’s protocol. A NanoVue UV–vis spectrophotometer (GE Healthcare Bio-Science, Uppsala, Sweden) was used to quantify RNA by measuring the absorbance at 230, 260, and 280 nm. An absorbance ratio of OD260/280 and OD260/230 was taken into account for assessing the purity of all RNA samples. The integrity of RNA was checked by 1 % agarose gel electrophoresis. After total RNA extraction and DNase I treatment, magnetic beads with Oligo (dT) were used to isolate the mRNA. After mixing the fragmentation buffer, the mRNA was fragmented into short fragments. cDNA was then synthesized using the mRNA fragments as templates. Short fragments were purified and suspended with No. 15012547 elution buffer in TruSeq™ RNA sample prep kit (Catalog No. RS-930-2001) (Illumina, San Diego, CA, USA) for end reparation and single nucleotide A (adenine) addition. The short fragments were then connected with No. 15013688–15013695 adapters in TruSeq™ RNA sample prep kit. Suitable fragments are selected for the PCR amplification as templates. During the quality control steps, Agilent 2100 Bioanaylzer (Agilent Technologies, Palo Alto, CA, USA) and ABI StepOnePlus Real-Time PCR System (ABI, New York, NY, USA) were used for quantification and qualification of the sample library. Finally, the library was sequenced using Illumina HiSeq™ 2000 (Illumina).

### Bioinformatics analysis

Base calling was used to transform image data output from the sequencing machine into sequence data. These data are called raw reads and are stored in the fastq format. Raw reads were filtered to remove reads containing adapters, unknown or low-quality bases. The data used in this publication have been deposited in the NIH Short Read Archive database (http://www.ncbi.nlm.nih.gov/sra) and are accessible through SRA accession number SRP030466 (Alias: PRJNA221355). The short reads assembly program Trinity (Grabherr et al. 2011) was used for de novo transcriptome assembly.

The sequences output by Trinity were termed unigenes. When multiple samples from the same species are sequenced, unigenes from each sample’s assembly can be further processed for sequence splicing and redundancy removal with sequence clustering software to generate the longest possible non-redundant unigenes, which are called all-unigenes. After gene family clustering, the unigenes were divided into two classes. One class is clusters, which have the prefix CL followed by the cluster ID. In clusters, the similarity among the unigenes is >70 %. The other class is singletons, which simply have the prefix unigene.

BLASTX alignment (e-value <0.00001) between unigenes and protein databases including non-redundant protein database (NR) (ftp://ftp.ncbi.nih.gov/blast/db/nr), Swiss-Prot (http://www.ebi.ac.uk/swissprot/), Kyoto Encyclopedia of Genes and Genomes (KEGG) (http://www.genome.jp/kegg/), and clusters of orthologous groups (COG) (http://www.ncbi.nlm.nih.gov/COG/) were performed. The sequence directions of unigenes were decided by the best alignment results in these databases. If different databases generated conflicting results, the sequence direction of a unigenes was decided in priority order of NR, Swiss-Prot, KEGG, and COG. When a unigene could not be aligned to any of the above databases, the software ESTScan (Iseli et al. 1999) was used to decide its sequence direction.

### Unigene function annotation

After aligning the unigene sequences to the protein databases, they were aligned to nucleotide databases nt (e-value <1.0E − 5) by blastn. Proteins, along with their protein functional annotations, that had the highest sequence similarity with the given conceptual translation of the unigenes were retrieved. KEGG annotation permitted pathway annotation of unigenes. The unigenes were also aligned to the COG database to predict and classify the possible functions of the unigenes.

### Unigene GO classification

The Blast2GO program (Conesa et al. 2005) was used to obtain GO annotation of the unigenes. To understand the distribution of the functions of the genes in *P. fortunei* at the macro level, GO functional classification was performed for all unigenes using the WEGO software (Ye et al. 2006).

### Protein coding sequence prediction

The unigenes were initially aligned by BLASTX (e-value <1.0E − 5) to protein databases in the priority order of NR, Swiss-Prot, KEGG, and COG. Proteins whose ranks were highest in the blast results were retrieved to decide the coding sequences (CDSs) of the unigenes. The CDS were then translated into amino acid sequences with the standard codon table. Thus, both the nucleotide sequences (5′–3′) and amino sequences of the unigene CDSs were acquired. ESTScan (Iseli et al. 1999) was used to scan unigenes that could not be aligned to any database, producing nucleotide sequence (5′–3′) direction and amino sequence of the predicted coding region.

### Unigene expression difference analysis

The fragments per kb per million fragments (FPKM) method was used to identify differentially expressed genes (DEGs) (Mortazavi et al. 2008). A rigorous algorithm was developed to identify genes differentially expressed between two samples by Audic and Claverie (1997). The threshold *p*-value in multiple tests was determined using the false discovery rate (FDR) method (Broberg 2005). A threshold FDR <0.001 and an absolute value of log2Ratio >1 were used to judge the significance of differences in gene expression. DEGs were then subjected to GO functional analysis and KEGG pathway analysis.

### Gene ontology functional enrichment analysis for DEGs

GO analysis provides GO functional classification annotation functional enrichment analysis for DEGs. First, all the DEGs were examined for each term of the Gene Ontology database (http://www.geneontology.org/), and the gene numbers for each GO-term were calculated. To identify significantly enriched GO terms in DEGs, a hypergeometric test was used. The calculated *p*-value was subjected to Bonferroni correction, taking a corrected *p*-value ≤0.05 as a threshold. GO terms fulfilling this condition were defined as significantly enriched GO terms in DEGs in the context of the whole transcriptome background. This analysis identified the main biological functions associated with the DEGs. The GO functional enrichment analysis also integrated the clustering analysis of expression patterns to easily determine the expression patterns of DEGs annotated to the given GO-term.

### KEGG pathway analysis for DEGs

Pathway enrichment analysis retrieved significantly enriched pathways associated with DEGs in the context of the whole transcriptome background. The formula for calculating the *p*-value was similar to that used in the GO analysis. The *Q*-value was defined as the FDR analog of the *p*-value. After multiple testing correction, we chose pathways with *Q*-values ≤0.05 as significantly enriched in DEGs.

### Quantitative real-time PCR analysis of DEGs

The RNA samples from the leaves of the PF2W75, PF4W75, PF2W25-12D, and PF4W25-12D samples were extracted with Trizol (Sangon, Shanghai, China). The RNA was then precipitated with isopropanol. Purified and concentrated RNA was denatured and first-strand cDNAs for all the samples were synthesized using a PrimeScript RT reagent Kit (Takara, Dalian, China). Potential genes related to drought response were chosen. The primers were designed with Beacon Designer, version 7.7 (Premier Biosoft International, Ltd., Palo Alto, CA, USA). The cDNAs were then amplified in a Bio-Rad CFX96TM Real-Time System (Bio-Rad, Hercules, CA, USA) with SYBR Premix Ex Taq TM II (Takara, Dalian, China). The following PCR parameters were used: 50 °C for 2 min, 95 °C for 30 s followed by 40 cycles of 94 °C for 15 s and 60 °C for 1 min. Three replicates were analyzed for each gene. The average threshold cycle (Ct) was normalized and the relative expression changes were calculated using the 2^−△△Ct^ method. The 18S rRNA of *Paulownia* was chosen as an internal reference gene for normalization.

## Results

### Comparative studies on physiological responses of diploid and tetraploid *Paulownia* to drought stress

**Fig. 1:**
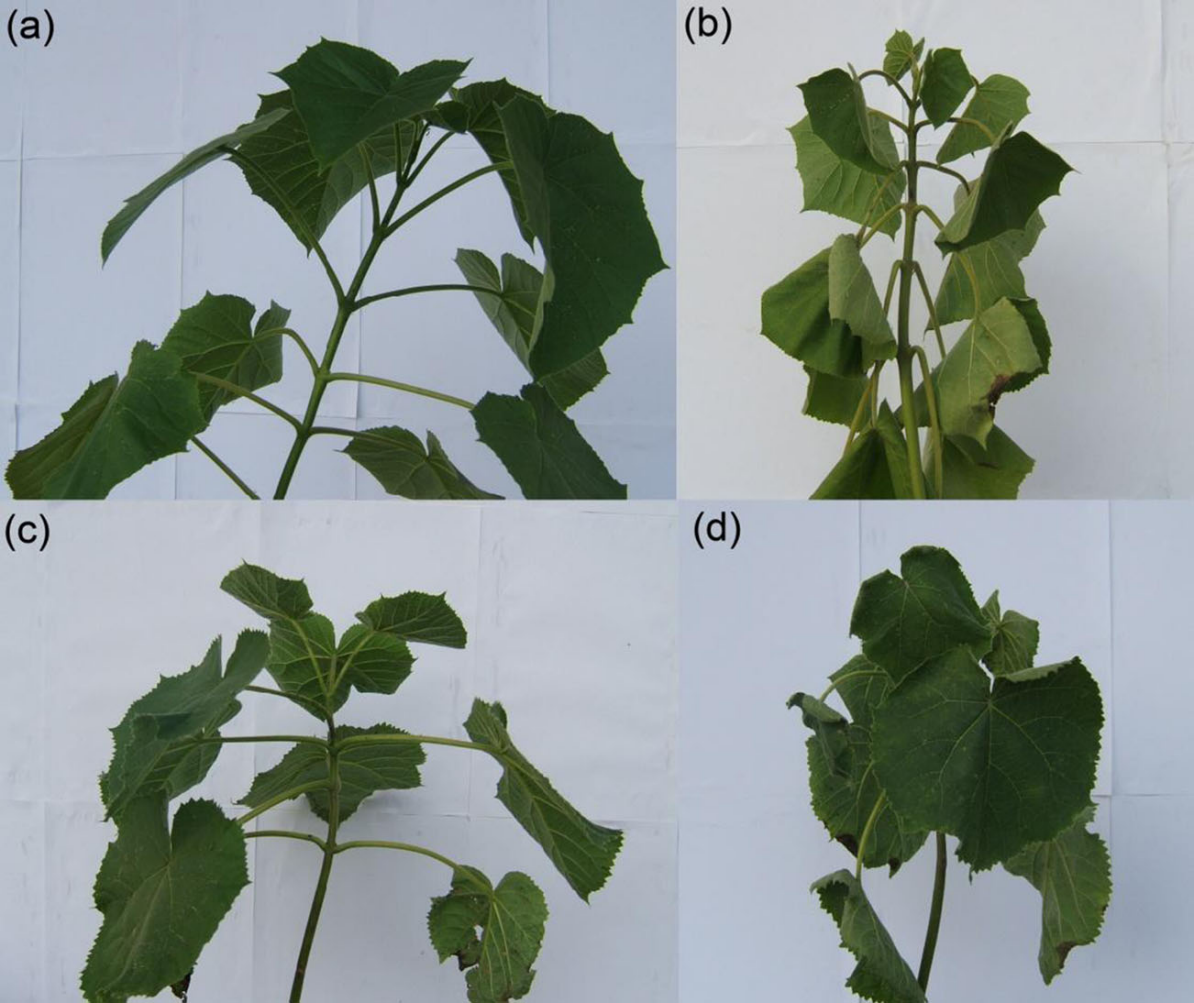
Tissues used for the transcriptome analysis. **a** *PF2W75* well-watered diploid, **b** *PF2W25-12D* 12 days drought-treated diploid, **c** *PF4W75* well-watered tetraploid, **d** *PF4W25-12D* 12 days drought-treated tetraploid

**Fig. 2:**
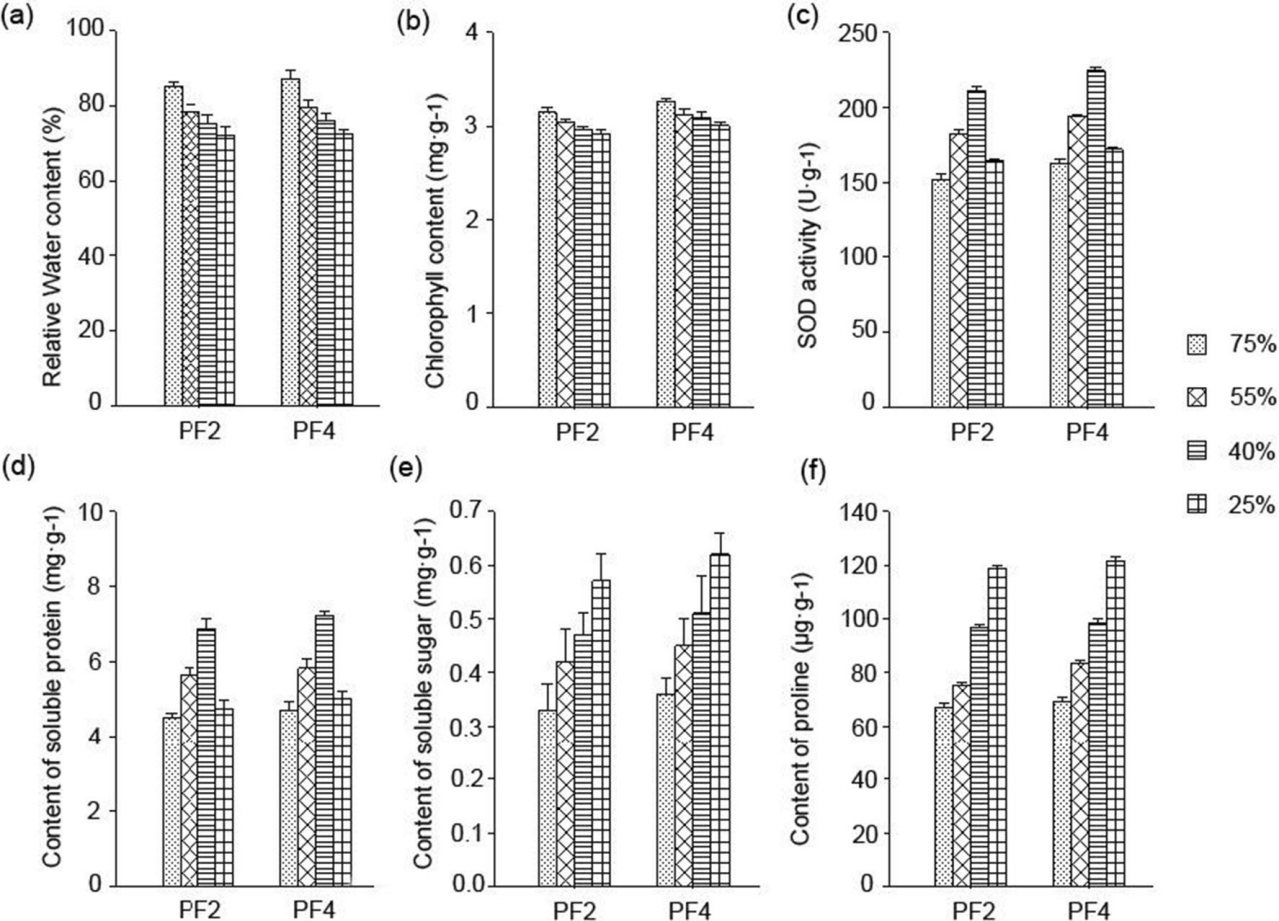
Effects of drought stress on *P. fortunei* physiology. *PF2* diploid *P. fortunei*, *PF4* autotetraploid *P. fortunei*. **a** Effect of drought stress on leaf relative water content. **b** Effect of drought stress on leaf chlorophyll content. **c** Effect of drought stress on leaf SOD activities. **d** Effect of drought stress on leaf soluble protein content. **e** Effect of drought stress on leaf soluble sugar content. **f** Effect of drought stress on leaf proline content

With 25 and 75 % relative soil water contents, the physiological responses of tetraploids and diploid *Paulownia* plants to drought stress tolerance were studied. In the tetraploid and diploid *Paulownia* plants, the changing trends of leaf physiological and biochemical indexes were consistent with aggravation of drought stress (Fig. 1). The water and chlorophyll contents of the leaves decreased during drought stress. The water and chlorophyll contents of the drought-treated plants leaves were higher than those of the well-watered plants in both the tetraploids and diploids. The SOD activity and soluble protein content initially increased and then decreased. The soluble sugar and proline contents increased during drought stress, with higher levels in the tetraploids than in the diploids (Fig. 2) (Zhang et al. 2013).

### Solexa sequencing and sequence assembly

**Fig. 3:**
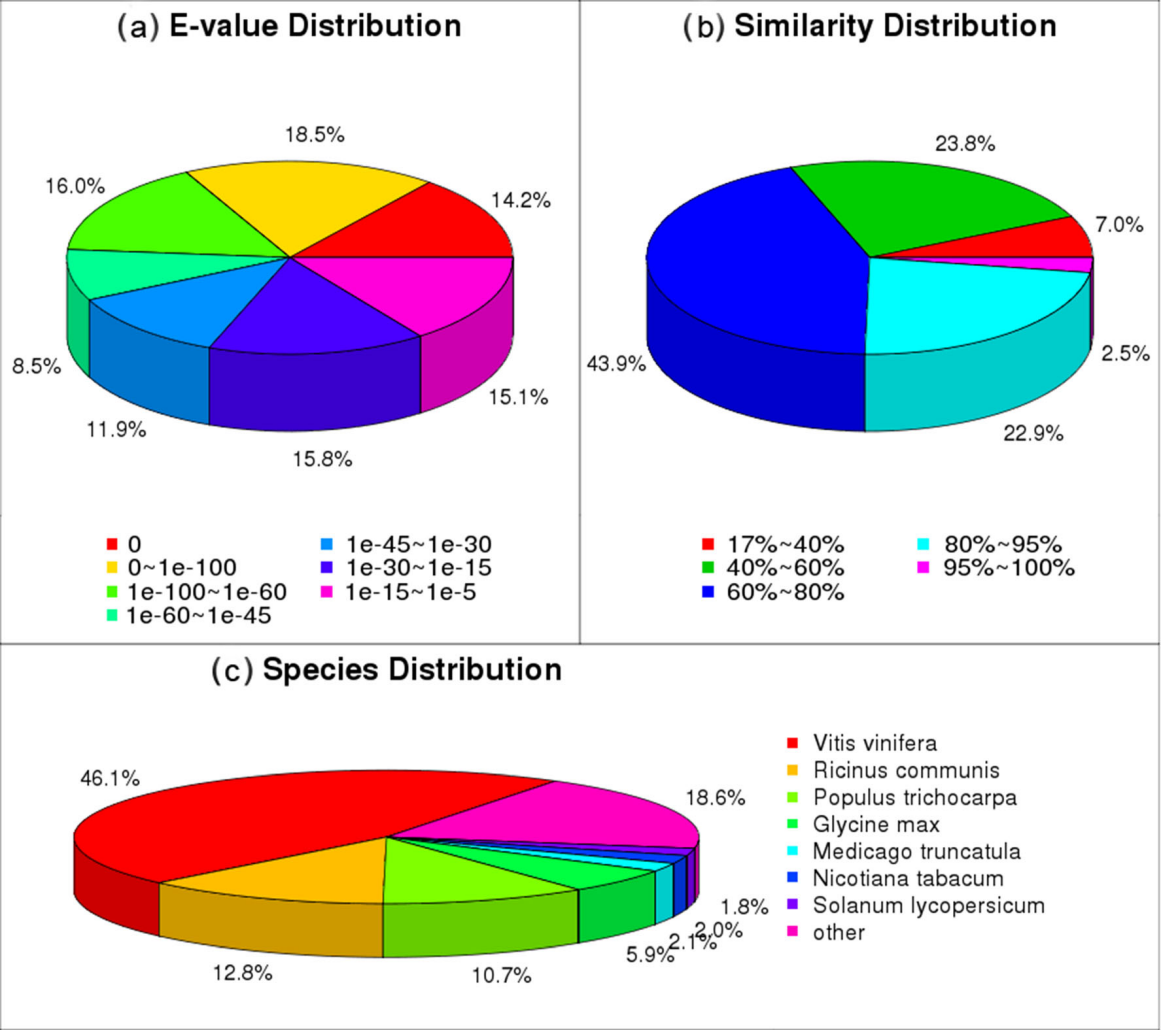
Characteristics of homology search of Illumina sequences against the nr database. **a** The e-value distribution of BLAST hits for each unique sequence with a cutoff e-value of 1.0E − 5. **b** Similarity distribution of the top BLAST hits for each sequence. **c** Species distribution is shown as a percentage of the total homologous sequences with an e-value of at least 1.0E − 5

Table 1 summarizes the results of Solexa sequencing and the distribution of clean tags. A total of 296,101,954 raw reads were sequenced in the library of four samples. After filtering out low-quality data with Q20 percentages up to 97.5 %, 266,700,100 clean reads with 24,003,009,000 nt of clean nucleotides remained in the total library. The PF2W25-12D library showed the highest ratio of clean reads to total reads. These short reads were assembled into 605,754 contigs using Trinity, with a mean contig length of 310 bp. These contigs were further assembled into unigenes. After further processing for sequence splicing and redundancy removal, 110,586 unigenes were generated, with a mean length of 922 bp. The N50 (medium of the length) of the all-unigene libraries was 1,523 bp. Among these unigenes, 50,890 were shorter than 500 bp, and 36,347 were longer than 1,000 bp. The length distribution of these contigs and unigenes is shown in Supplementary Fig. S1. The gap distribution of unigenes was analyzed to identify the data quality. No unigenes showed any gaps, demonstrating the high quality of the assembly. The e-value distribution and similarity distribution of the all-unigenes against nr database are shown in Fig. 3a, b, respectively. Experiment pipeline of sequencing is shown in Supplementary Fig. S2a.

**Table 1:** Overview of the sequencing and assembly

	PF2W75	PF4W75	PF2W25-12D	PF4W25-12D
Statistics of data production				
Number of clean reads	64,267,788	67,074,340	66,552,344	68,805,628
Total nucleotides (nt)	5,784,100,920	6,036,690,600	5,989,710,960	6,192,506,520
Q20 percentage (%)	97.45 %	97.45 %	97.57 %	97.47 %
N percentage	0.00 %	0.00 %	0.00 %	0.00 %
GC percentage (%)	46.92 %	47.27 %	45.68 %	46.32 %
Contigs				
Number of contigs	139,372	141,810	162,224	162,348
Total nucleotides (nt) in contigs	43,683,972	45,573,011	49,377,829	48,777,613
Average length of contigs (nt)	313	321	304	300
Length of N50 (bp)	503	472	461	456
Unigenes				
Number of unigenes	76,950	94,034	97,232	92,772
Total nucleotides (nt) in unigenes	53,834,403	54,457,691	74,391,848	65,686,297
Length of N50 (bp)	932	863	932	863
Average length of unigenes (bp)	700	579	765	708
All-unigenes				
Number of all-unigenes	110,580			
Total nucleotides (nt) in all-unigenes	101,972,540			
Length of N50 (bp)	1523			
Average length of all-unigenes (bp)	922

### Annotation of the predicted proteins

The full genome sequence for *P. fortunei* is not available. All-unigene sequences were first aligned by BLASTX to protein databases including NR, Swiss-Prot, KEGG, and COG, with a cutoff e-value of 1.0E − 5, and proteins whose sequence similarities were the highest were retrieved. A total of 72,586 distinct sequences, which comprised 65.6 % of all the unigenes, had a match with the known proteins in the database (Supplementary Table S1). The majority of sequences (about 46.1 %) had strong homology with *Vitis vinifera* (Fig. 3c). Among the matched unigenes, 12.8 % has a best match to sequences from *Ricinus communis*, followed by *Populus trichocarpa* (10.7 %), *Glycine max* (5.9 %), *Medicago truncatula* (2.1 %), *Nicotiana tabacum* (2.0 %), *Solanum lycopersicum* (1.8 %), and other plant species.

### Unigene function annotation

**Fig. 4:**
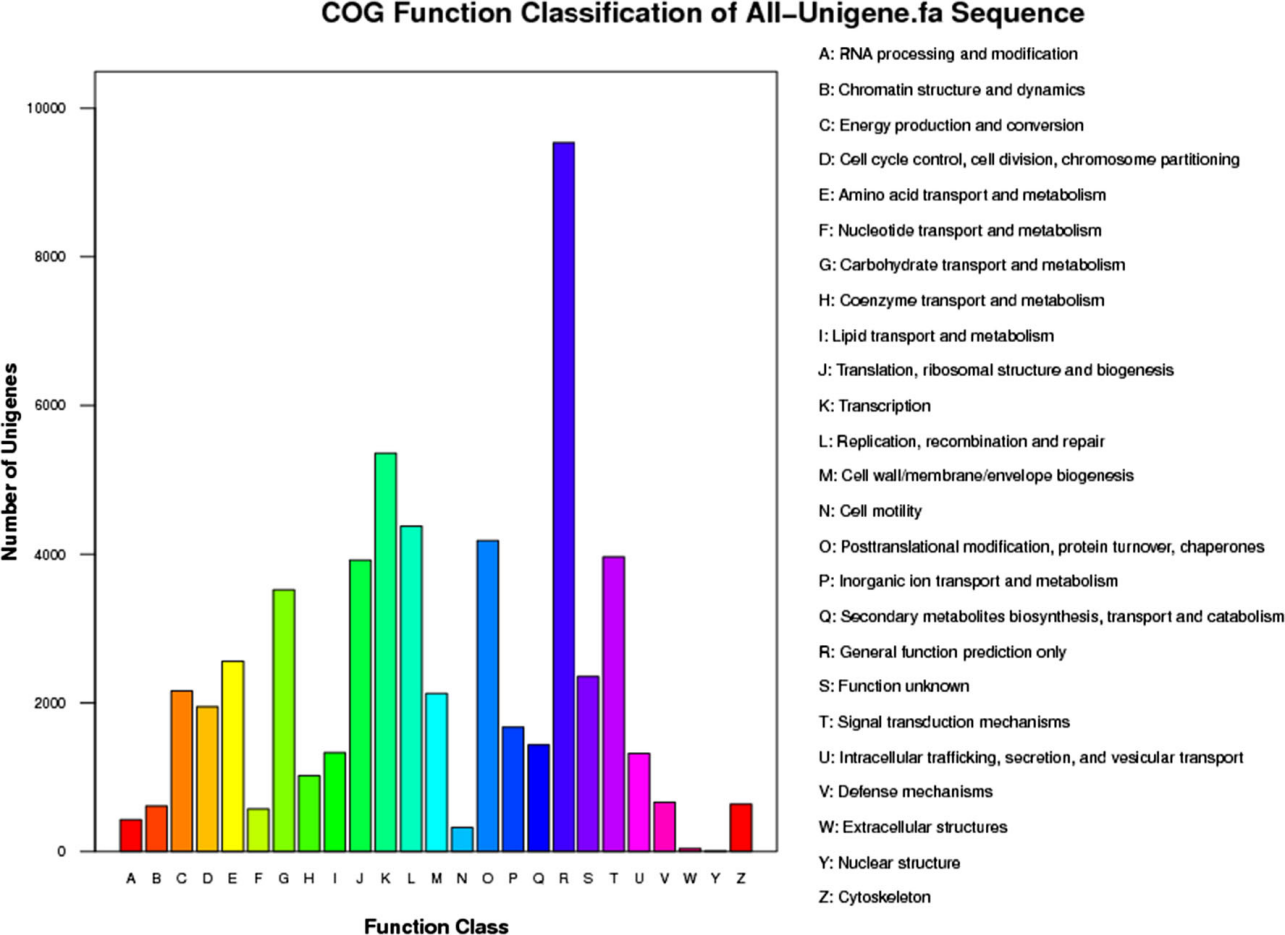
Classification of the clusters of orthologous groups (*COG*) (http://www.ncbi.nlm.nih.gov/COG/) for the transcriptome of *P. fortunei* all-unigenes; 29,510 unigenes (26.69 % of the total) were annotated and divided into 25 specific categories

To predict and classify the possible functions of the unigenes, COG assignments were used. Based on sequence homology, 29,510 unigenes, which comprised 26.69 % of all the unigenes, were annotated and could be divided into 25 specific categories (Fig. 4). The general function category containing 9,532 unigenes (8.62 %) was the largest, followed by transcription (5,359, 4.85 %), replication, recombination and repair (4,376, 3.96 %), posttranslational modification, protein turnover, and chaperones (4,179, 3.78 %), signal transduction mechanisms (3963, 3.58 %), translation, ribosomal structure, and biogenesis (3,918, 3.54 %), and carbohydrate transport and metabolism (3,519, 3.18 %). The smallest group had only six unigenes (0.0054 %), which belonged to the group of the nuclear structure. Three ontologies were divided for gene ontology (GO) analysis of the unigenes: molecular function, cellular component, and biological process. A total of 59,572 unigenes (53.87 % of total) were categorized into 57 functional groups. Cell and cell part were the two largest groups, both containing 47,808 unigenes. In the cellular component groups’ virion and virion part, only two unigenes for each were predicted (Supplementary Fig. S3).

### Unigene metabolic pathway analysis

The KEGG annotation system was used to conduct the unigene metabolic pathway analysis. A total of 44,154 unigenes, which comprised 39.93 % of all the unigenes, were mapped to 128 KEGG pathways. The metabolic pathway group, which comprised 10,364 unigenes (23.47 %), represented significantly more unigenes than any other pathways, such as biosynthesis of secondary metabolites (11.38 %), plant–pathogen interaction (5.61 %), plant hormone signal transduction (4.92 %), spliceosome (3.96 %), RNA transport (3.78 %), endocytosis (3.37 %), and glycerophospholipid metabolism (3.18 %) (Supplementary Table S2). Experiment pipeline of bioinformatics analysis is shown in Supplementary Fig. S2b.

### Transcripts encoding specific genes associated with the drought response

**Fig. 5:**
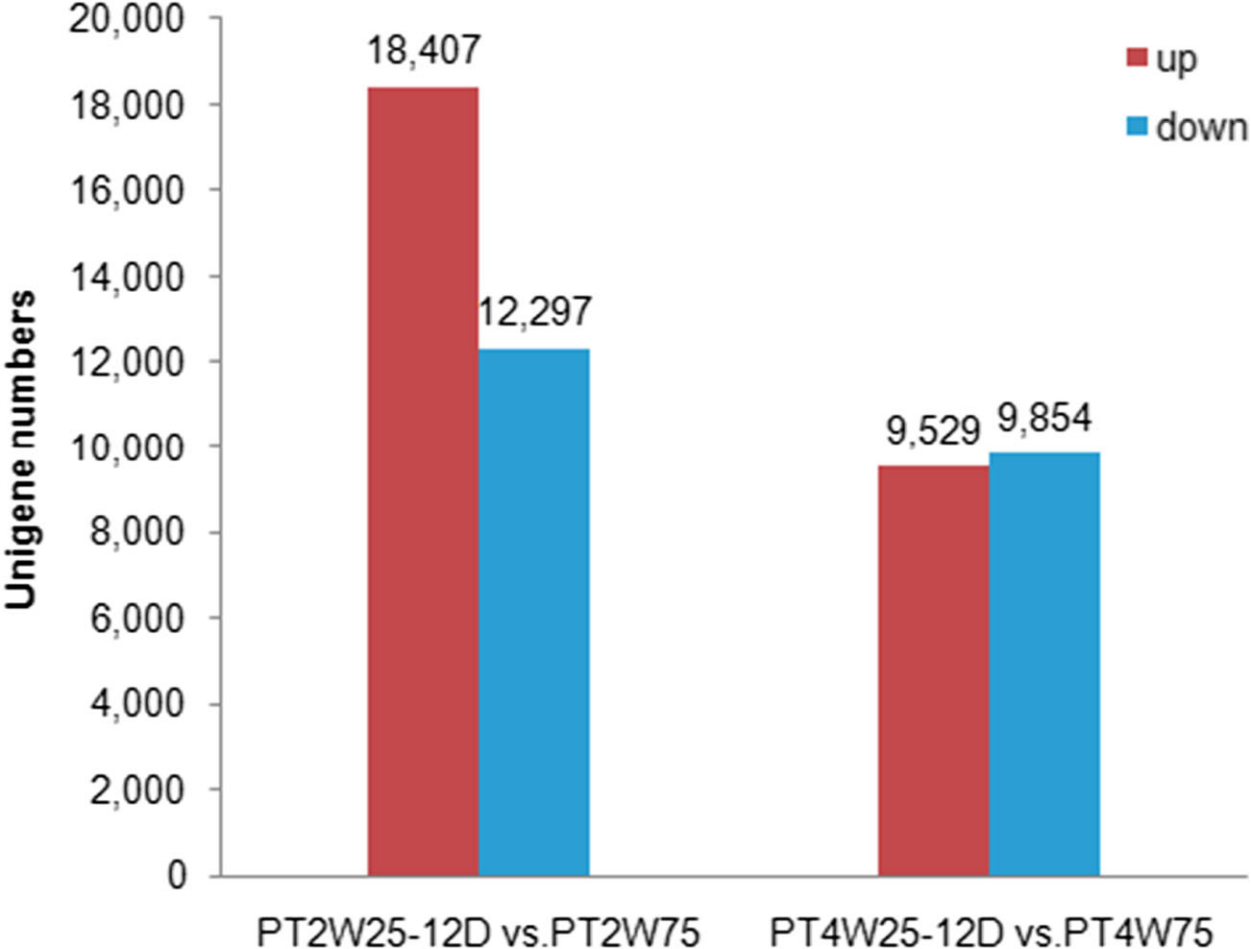
Statistics of differentially expressed genes in each pairwise comparison. *Red bars* represent the up-regulated genes, while *blue bars* represent the down-regulated ones. *PF4W25-12D* 12 days drought-treated tetraploid, *PF4W75* well-watered tetraploid, *PF2W25-12D* 12 days drought-treated diploid, *PF2W75* well-watered diploid

The four samples were evaluated in two pairwise comparisons: PF2W25-12D vs. PF2W75 and PF4W25-12D vs. PF4W75. Genes found to have significant expression differences in each comparison were identified (Fig. 5). The results suggested that in the PF2W25-12D vs. PF2W75 comparison, the expression of 30,704 genes was significantly different (Supplementary Table S3). Among these genes, 18,407 were up-regulated and 12,297 were down-regulated. Among the top ten up-regulated unigenes, only three could be aligned to genes encoding proteins with known functions in GenBank. Among the top ten down-regulated genes, five could be identified (Supplementary Table S4). In the comparison of PF4W25-12D and PF4W75, 19,383 DEGs were identified, among which 9,529 were up-regulated and 9,854 were down-regulated. After that, 10,383 genes consistently differentially expressed in both of the two comparisons were retrieved (Supplementary Table S5). The molecular functions of the 5,483 common DEGs are listed in Supplementary Table S6.

### Comparison of gene expression profiles among the different samples

DEGs were further characterized by GO classification into three groups: biological process, cellular component, and molecular function. The results of each comparison showed high consistency with genes involved in cellular component, which mainly concentrated in the cell category (15,479 genes in PF2W25-12D vs. PF2W75; 9,623 genes in PF4W25-12D vs. PF4W75) and cell part category (15,479 genes in PF2W25-12D vs. PF2, 9,623 genes in PF4W25-12D vs. PF4W75). In the comparison of PF2W75 and PF2W25-12D, 12,648 and 12,168 biological process genes were involved in cellular process and metabolic process, respectively. In the comparison of PF4W25-12D vs. PF4W75, the numbers were 8,099 and 7,910 (PF4W25-12D vs. PF4W75). Finally, 9,915 genes (PF2W25-12D vs. PF2W75) and 6,312 genes (PF4W25-12D vs. PF4W75) were involved in catalytic activity (Supplementary Figs. S4 and S5). In the KEGG analysis, the most number of DEGs were related to metabolic pathways in the two comparisons. The number of genes differently expressed was significantly higher than in the other pathways (Supplementary Tables S7 and S8). The KEGG analysis of the 5,162 common DEGs in both comparisons is listed in Supplementary Table S9.

### Confirmation of candidate drought response genes by qRT-PCR

**Fig. 6:**
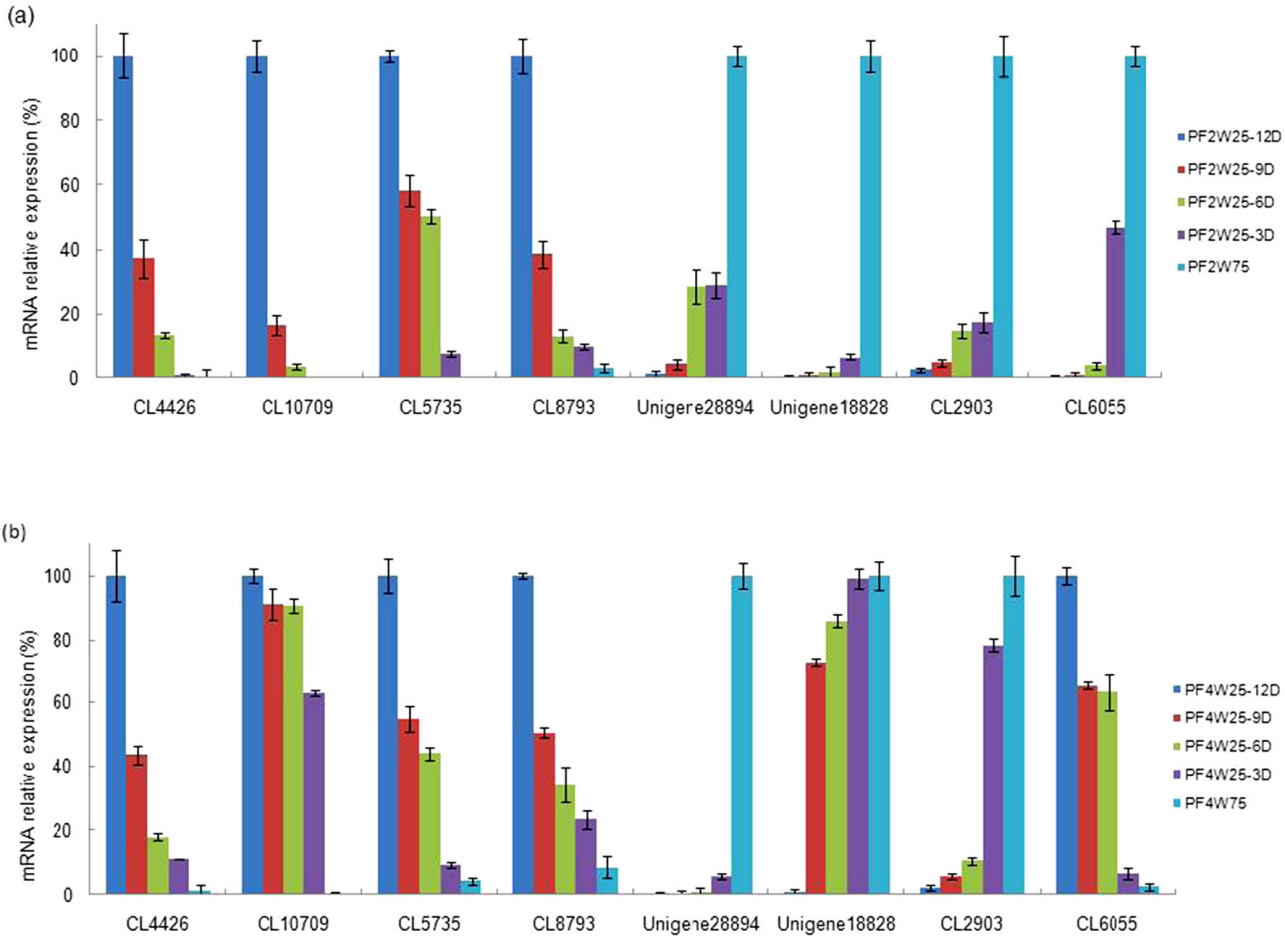
Quantitative RT-PCR analysis of candidate drought response genes. *CL4426* a peptide/nitrate transporter, *CL10709* a dehydrin, *CL5735* a flavonoid glycosyltransferase, *CL8793* a glycine-rich protein precursor, *Unigene28894* an alcohol dehydrogenase, *Unigene18828* a chlorophyll A/B binding protein, *CL2903* a disease resistance response protein, *CL6055* an auxin-induced protein X10A. 18S rRNA was used as the internal reference gene. For each group, the highest expression level was considered as 100 %, and other samples were normalized accordingly. Standard error of the mean for three technical replicates is represented by the *error bars*. **a** *PF2W25-12D* 12 days drought-treated diploid, *PF2W25-9D* 9 days drought-treated diploid, *PF2W25-6D* 6 days drought-treated diploid, *PF2W25-3D* 3 days drought-treated diploid, *PF2W75*, well-watered diploid. **b** *PF4W25-12D* 12 days drought-treated tetraploid, *PF4W25-9D* 9 days drought-treated tetraploid, *PF4W25-6D* 6 days drought-treated tetraploid, *PF4W25-3D* 3 days drought-treated tetraploid, *PF4W75* well-watered tetraploid

To confirm the reliability of Solexa/Illumina sequencing technology, eight genes from both diploid and tetraploid samples were randomly selected for quantitative RT-PCR assays. The results showed that except the auxin-induced protein X10A in tetraploid samples, the rest were consistent between the quantitative real-time PCR (qRT-PCR) and the transcriptome analyses. In both the tetraploid and diploid *Paulownia* plants, the changing trends of gene expression were consistent with aggravation of drought stress (Fig. 6). All the primers used for the qRT-PCR analysis, potential gene functions, and amplicon sizes are shown in Table 2. For the gene which showed the inconsistency between qRT-PCR and transcriptome, it was likely attributable to the fact that transcriptome was more sensitive in the detection of low-abundant transcripts and small changes in gene expression than qRT-PCR method. The results indicated the validity of the sequencing method.

**Table 2:** Primers of quantitative RT-PCR analysis of candidate drought response genes

Potential gene function	Size (bp)	Primer	Sequence
Peptide/nitrate Transporter	912	CL4426.Contig1	GGCTGGTCTAGTTAATGAT
		CL4426.Contig1-r	AATGGTACAAGTATAAGAGAAC
Dehydrin	1041	CL10709.Contig1	TGGCTTATTATTACAACTATT
		CL10709.Contig1-r	GAGTGATGGAGAAGATTA
Flavonoid Glycosyltransferase	1700	CL5735.Contig1	GTGATAAGTGGACCAACAG
		CL5735.Contig1-r	AGACGAGTAGAAGGATTGAA
Glycine-rich protein precursor	585	CL8793.Contig1	TAGCAGAGGTGTGAGAGT
		CL8793.Contig1-r	GTTCAACAGCAACATTACTACT
Alcohol dehydrogenase	1701	Unigene28894	AGTTATCTCCATCTATCAG
		Unigene28894-r	ATCAACATATCTACAATAAGG
Chlorophyll A/B binding protein	1016	Unigene18828	CCGATTCGTTCAATACACAT
		Unigene18828-r	AACTTGGCAACTCACTTG
Disease resistance response protein	714	CL2903.Contig2	GATGTAGACCTGGAACTT
		CL2903.Contig2-r	CACCCTTACTGAAATGAC
Auxin-induced protein X10A	1080	CL6055.Contig1	CAACTCCAAATACCCACCTT
		CL6055.Contig1-r	AAACCTCGTATCCTACCCATA

*r* reverse primer

## Discussion

Until recently, little molecular genetics and genomic research has focused on the family *Paulownia* spp. The lack of genetic information on this plant represents a large obstacle to studying the mechanisms of its ability to resist water stress. Analysis of the *Paulownia* transcriptome and its expression profile data is essential to extending the genetic resources for this species. Using the RNA-seq technique, the present study identified a series of genes whose expression is altered between drought-treated and well-watered samples of *P. fortunei* as well as identifying several associated pathways.

### Morphological features of the drought-treated and well-watered samples

The drought-treated (PF2W25-12D and PF4W25-12D) and well-watered samples (PF2W75 and PF4W75) showed significant variations in leaf structure under control and drought stress. The leaves in PF2W25-12D and PF4W25-12D showed adaptive phenomena such as inward rolling and smaller size of the leaves, which would limit water loss by evaporation through restricting the exposed leaf surface. The same phenomenon under drought stress has been reported previously (Galmes et al. 2013; Ashoub et al. 2013). Thus, leaves play an important role in preserving the physiological normal state of plants during drought stress.

### Genes encoding proteins that function in drought tolerance

Based on their biological functions, the drought-induced genes were associated with proteins that function in response to drought. Proteins involved in carbohydrate metabolism, such as sucrose, hexose, pentose, and sugar alcohols, comprised a large part. It is reported that the accumulation of nonstructural sugars strongly correlates with osmotic stress tolerance (Oliver et al. 2010). This could be explained by the fact that sugars permit the cell components to tolerate desiccation, which protects the plant against drought. Among the sugars, starch and trehalose are the storage carbohydrates that are mostly involved in this process. This group also includes genes involved in the detoxification of aldehydes through alcohol metabolism (Supplementary Table S9). This is in line with the observed accumulation of ethanol in conifer seedlings under water deficiency (Manter and Kelsey 2008). Within the group of genes encoding proteins that function in drought tolerance, the ones related to amino acids (e.g., proline), lipids, and fatty acid metabolism, which could be involved in the synthesis and accumulation of compatible solutes during water stress, are also included. The induction of methionine synthase transcripts were observed in both differentially expressed accessions. Increases in methionine synthase protein content under salt stress in barley (Narita et al. 2004) and in the roots of wild watermelon under water stress have also been reported (Yoshimura et al. 2008). However, another study showed that genes encoding enzymes of methionine synthesis showed no significant differences between drought-tolerant and drought-sensitive samples (Xu and Huang 2010). Changes of various free amino acids have been found in *Arabidopsis* and rice plants under several abiotic stresses (Ndimba et al. 2005; Yan et al. 2005). The activation of methionine synthase is an initial response to drought because increased flux through the pathway provides a source of methyl groups for secondary metabolism compounds that provide adaptive advantages to samples. Thus, the increase or maintenance of high levels of methionine synthase may reflect more active methylation and osmoregulant metabolism.

Another important group of genes encode transporter proteins. Transport processes are very important in the mobilization and accumulation of solutes and hormones. Furthermore, these processes play an important part in cell detoxification pathways during adaptation to drought. Thus, sugar transporters, such as hexose transporters, are involved in the modification of osmotic pressure (Wanke and Kolukisaoglu 2010). Anion, sodium, and solute–cation symporter transmembrane transporters, calcium–cation, and anion–anion antiporters deserve special mention. These channels, whose expression is induced by water stress, mediate water flux, which can maintain proper water balance within and outside both the plasma membrane and the vacuole membrane. Some studies have been done on this protein family in woody species, and their role during water stress has been suggested (Almeida-Rodriguez et al. 2010; Berta et al. 2010). Other transporters of secondary metabolites, organic acids, l-glutamate, carboxylic acid, acidic amino acid, cationic amino acid, arginine, l-lysine, myo-inositol, dicarboxylic acid, and auxin all belong to this group.

### Genes encoding regulatory proteins in response to drought

Regulatory proteins are known to play an important part in plant response and adaption to abiotic stresses. Transcription factors, such as WRKY and MYB, and regulatory proteins such as some calcium-binding proteins, nucleic acid-binding proteins, serine/threnine proteins, zinc finger proteins known for leaf development under drought stress were highly expressed in PF2W25-12D and PF4W25-12D samples. Some secondary metabolites, such as flavonoids, were also up-regulated in the two accessions. In a report about the physiological and molecular responses of *Populus canadensis* to drought, the stress was applied to young rooted cuttings by PEG 6000 application over 30 days. Genes encoding transcription factor MYB2 was identified (Caruso et al. 2008). Chang et al. (1996) identified several water deficit stress-inducible genes from *Pinus taeda* L. Two putative drought-induced proteins were involved in calcium and nucleic acid binding. In the cloned and identified drought-induced genes in maritime pine roots, serine/threnine protein kinase, RAS-related protein, and ring zinc finger protein also acted as regulatory elements response to drought (Dubos and Plomion 2003). Several studies have also indicated that flavonoids increase in plants under stress (Yang et al. 2007a), which is in accordance with the results of the current study.

### Genes involved in growth and development in response to drought

**Fig. 7:**
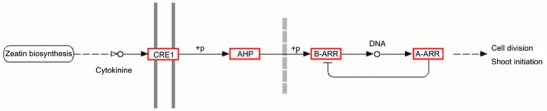
Zeatin biosynthesis pathway in *P. fortunei*. Up-regulated expressed genes in both PF2W25-12D and PF4W25-12D are in *red boxes. Double solid line* plasma membranes of the leaf of *P. fortunei. Dashed line* nuclear membranes of the leaf cell

A part of the identified DEGs in the present study encoded cell components and various hormones such as auxin and cytokinin, which serve as regulators of plant growth and development. In the research of *P. taeda* L. (Chang et al. 1996), putative drought-induced proteins which are involved in lignin biosynthesis and cell wall modification were reported. The well-described ABA-dependent pathway in plants’ response to drought uses ABA as the main phytohormone (Christmann et al. 2006). ABA is known to act as an endogenous messenger in drought response and adaption processes. It is induced by water deficit, which triggers major alterations to gene expression patterns in plant cells. Besides, it contributes to other important processes like plant growth, development, or seed dormancy (Hirayama and Shinozaki 2007). In the present study, some of the DEGs were annotated as being involved in crosstalk among ABA and other important phytohormones, such as ethylene, auxin, zeatin, and brassinosteroid. Ethylene was reported to be involved in almost all kinds of abiotic stresses, including drought (Lu et al. 2007). A series of genes encoding enzymes in the zeatin biosynthesis pathway was found to be elevated in both PF2W25-12D and PF4W25-12D accessions (Fig. 7). A research on the effect of water deficit on the transcriptome of the *Populus* cambial region has also reported the same results (Berta et al. 2010). How drought stress is perceived by the ABA signal is still largely unknown, which needs to be investigated further. In our study, a series of ABA synthesis-related enzymes and transporters was found in the drought-induced samples. It has been reported that water-scarce conditions can trigger an immediate hydraulic signal, which may activate ABA biosynthesis over a great distance (Christmann et al. 2007). The signal can then activate enzymes like cytochrome P450 to catalyze ABA synthesis only a few minutes after the signal is received (Okamoto et al. 2009). ABA transporters are also important during drought response processes. Recent studies have shown that on perception of a signal, ABA is synthesized primarily in vascular tissues and then exported to other cells in the plant. The family of ATP-dependent ABC transporters can stimulate the absorption of ABA. This mechanism is useful in the rapid distribution of ABA hormone to the surrounding tissues (Kang et al. 2010; Kuromori et al. 2010).

### Plasticity of the genes identified in response to drought

Although certain genes are regulated in a similar way among different plant species, there are many genes that are observed to have significantly different expression patterns among different species or, occasionally, in the same species under different experimental conditions (Stern et al. 2007; Narsai et al. 2010). Relatively low correspondence of the related genes may be partially explained by genome transcriptional reprogramming induced by drought stress being very plastic: multiple pathways may be activated in parallel in drought conditions and these pathways can confer drought tolerance independently or synergistically. It is also possible that the observed differences reflect different developmental stages, tissue types, or even experimental procedures. Some of the DEGs in this study did not show any similarity with sequences in the databases; therefore, they represent potentially novel proteins that are associated with the specific plant responses to water stress.

## Electronic supplementary material

Below is the link to the electronic supplementary material.

Fig. S1 (DOCX 24 kb)

Fig. S2 (DOCX 162 kb)

Fig. S3 (DOCX 120 kb)

Fig. S4 (DOCX 115 kb)

Fig. S5 (DOCX 115 kb)

Table S1 (XLSX 3296 kb)

Table S2 (XLSX 17 kb)

Table S3 (XLS 220 kb)

Table S4 (XLS 238 kb)

Table S5 (XLSX 3050 kb)

Table S6 (XLSX 66 kb)

Table S7 (XLS 39 kb)

Table S8 (XLS 44 kb)

Table S9 (XLSX 14 kb)

## References

[CR1] Adams KL, Cronn R, Percifield R, Wendel JF (2003). Genes duplicated by polyploidy show unequal contributions to the transcriptome and organ-specific reciprocal silencing. Proc Natl Acad Sci U S A.

[CR2] Almeida-Rodriguez AM, Cooke JE, Yeh F, Zwiazek JJ (2010). Functional characterization of drought-responsive aquaporins in *Populus balsamifera* and *Populus simonii* × *balsamifera* clones with different drought resistance strategies. Physiol Plant.

[CR3] Aranda I, Gil-Pelegrín E, Gascó A, Guevara MA, Cano JF, Miguel M, Ramírez-Valiente JA, Peguero-Pina JJ, Perdiguero P, Soto A, Cervera MT, Collada C, Aroca R (2012). Drought response in forest trees: from the species to the gene. Plant responses to drought stress.

[CR4] Ashoub A, Beckhaus T, Berberich T, Karas M, Bruggemann W (2013). Comparative analysis of barley leaf proteome as affected by drought stress. Planta.

[CR5] Audic S, Claverie JM (1997). The significance of digital gene expression profiles. Genome Res.

[CR6] Bae EK, Lee H, Lee JS, Noh EW (2009). Differential expression of a poplar SK2-type dehydrin gene in response to various stresses. BMB Rep.

[CR7] Bae EK, Lee H, Lee JS, Noh EW (2010). Isolation and characterization of osmotic stress-induced genes in poplar cells by suppression subtractive hybridization and cDNA microarray analysis. Plant Physiol Biochem.

[CR8] Bae EK, Lee H, Lee JS, Noh EW (2011). Drought, salt and wounding stress induce the expression of the plasma membrane intrinsic protein 1 gene in poplar (*Populus alba* × *P. tremula* var. glandulosa). Gene.

[CR9] Berta M, Giovannelli A, Sebastiani F, Camussi A, Racchi ML (2010). Transcriptome changes in the cambial region of poplar (*Populus alba* L.) in response to water deficit. Plant Biol.

[CR10] Broberg P (2005). A comparative review of estimates of the proportion unchanged genes and the false discovery rate. BMC Bioinforma.

[CR11] Caruso A, Chefdor F, Carpin S, Depierreux C, Delmotte FM, Kahlem G, Morabito D (2008). Physiological characterization and identification of genes differentially expressed in response to drought induced by PEG 6000 in *Populus canadensis* leaves. J Plant Physiol.

[CR12] Chang S, Puryear JD, Dias M, Funkhouser EA, Newton RJ, Cairney J (1996). Gene expression under water deficit in loblolly pine (*Pinus taeda*): isolation and characterization of cDNA clones. Physiol Plant.

[CR13] Chen J, Xia X, Yin W (2011). A poplar DRE-binding protein gene, PeDREB2L, is involved in regulation of defense response against abiotic stress. Gene.

[CR14] Christmann A, Moes D, Himmelbach A, Yang Y, Tang Y, Grill E (2006). Integration of abscisic acid signalling into plant responses. Plant Biol.

[CR15] Christmann A, Weiler EW, Steudle E, Grill E (2007). A hydraulic signal in root‐to‐shoot signalling of water shortage. Plant J.

[CR16] Conesa A, Gotz S, Garcia-Gomez JM, Terol J, Talon M, Robles M (2005). Blast2GO: a universal tool for annotation, visualization and analysis in functional genomics research. Bioinformatics.

[CR17] Costa GG, Cardoso KC, Del Bem LE, Lima AC, Cunha MA, de Campos-Leite L, Vicentini R, Papes F, Moreira RC, Yunes JA, Campos FA, Da Silva MJ (2010). Transcriptome analysis of the oil-rich seed of the bioenergy crop *Jatropha curcas* L. BMC Genomics.

[CR18] Dubos C, Plomion C (2003). Identification of water-deficit responsive genes in maritime pine (*Pinus pinaster* Ait.) roots. Plant Mol Biol.

[CR19] Fan GQ, Cao YC, Zhao ZL, Yang ZQ (2007). Induction of autotetraploid of *Paulownia fortunei*. Sci Silvae Sin.

[CR20] Gailing O, Vornam B, Leinemann L, Finkeldey R (2009). Genetic and genomic approaches to assess adaptive genetic variation in plants: forest trees as a model. Physiol Plant.

[CR21] Galmes J, Ochogavia JM, Gago J, Roldan EJ, Cifre J, Conesa MA (2013). Leaf responses to drought stress in Mediterranean accessions of *Solanum lycopersicum*: anatomical adaptations in relation to gas exchange parameters. Plant Cell Environ.

[CR22] Gimeno J, Gadea J, Forment J, Perez-Valle J, Santiago J, Martinez-Godoy MA, Yenush L, Belles JM, Brumos J, Colmenero-Flores JM, Talon M, Serrano R (2009). Shared and novel molecular responses of mandarin to drought. Plant Mol Biol.

[CR23] Grabherr MG, Haas BJ, Yassour M, Levin JZ, Thompson DA, Amit I, Adiconis X, Fan L, Raychowdhury R, Zeng Q, Chen Z, Mauceli E, Hacohen N, Gnirke A, Rhind N, di Palma F, Birren BW, Nusbaum C, Lindblad-Toh K, Friedman N, Regev A (2011). Full-length transcriptome assembly from RNA-Seq data without a reference genome. Nat Biotechnol.

[CR24] Guo LB, Qian Q, Zeng DL, Dong GJ, Teng S, Zhu LH (2004). Genetic dissection for leaf correlative traits of rice (*Oryza sativa* L.) under drought stress. Acta Genet Sin.

[CR25] Hamanishi ET, Campbell MM (2011). Genome-wide responses to drought in forest trees. Forestry.

[CR26] Hirayama T, Shinozaki K (2007). Perception and transduction of abscisic acid signals: keys to the function of the versatile plant hormone ABA. Trends Plant Sci.

[CR27] Iseli C, Jongeneel CV, Bucher P (1999) ESTScan: a program for detecting, evaluating, and reconstructing potential coding regions in EST sequences. ISMB. pp 138–147 10786296

[CR28] Kang JH, Wang JU, Lee M, Kim YY, Assmann SM, Martinoia E, Lee Y (2010). PDR-type ABC transporter mediates cellular uptake of the phytohormone abscisic acid. Proc Natl Acad Sci U S A.

[CR29] Krikorian AD (1988). *Paulownia* in China: cultivation and utilization. Econ Bot.

[CR30] Kuromori T, Miyaji T, Yabuuchi H, Shimizu H, Sugimoto E, Kamiya A, Moriyama Y, Shinozaki K (2010). ABC transporter AtABCG25 is involved in abscisic acid transport and responses. Proc Natl Acad Sci U S A.

[CR31] Liu J, Liu X (2011). Tutorial of plant physiology experiments.

[CR32] Lu W, Li Y (2012). Tutorials of plant physiological experiment.

[CR33] Lu PL, Chen NZ, An R, Su Z, Qi BS, Ren F, Chen J, Wang XC (2007). A novel drought inducible gene, ATAF1, encodes a NAC family protein that negatively regulates the expression of stress-responsive genes in *Arabidopsis*. Plant Mol Biol.

[CR34] Luo MH, Hu JY, Wu QG, Yang JT, Su ZX (2010) Effects of drought stress on leaf gas exchange and chlorophyll fluorescence of *Salvia miltiorrhiza*. Chin J Appl Ecol 21(3):619–623 20560316

[CR35] Lyons A, Race D (1993). Paulownia. Agroforestry: trees for productive farming.

[CR36] Manter DK, Kelsey RG (2008). Ethanol accumulation in drought-stressed conifer seedlings. Int J Plant Sci.

[CR37] Mortazavi A, Williams BA, McCue K, Schaeffer L, Wold B (2008). Mapping and quantifying mammalian transcriptomes by RNA-Seq. Nat Methods.

[CR38] Narita Y, Taguchi H, Nakamura T, Ueda A, Shi W, Takabe T (2004). Characterization of the salt-inducible methionine synthase from barley leaves. Plant Sci.

[CR39] Narsai R, Castleden I, Whelan J (2010). Common and distinct organ and stress responsive transcriptomic patterns in *Oryza sativa* and *Arabidopsis thaliana*. BMC Plant Biol.

[CR40] Ndimba BK, Chivasa S, Simon WJ, Slabas AR (2005). Identification of *Arabidopsis* salt and osmotic stress responsive proteins using two-dimensional difference gel electrophoresis and mass spectrometry. Proteomics.

[CR41] Okamoto M, Tanaka Y, Abrams SR, Kamiya Y, Seki M, Nambara E (2009). High humidity induces abscisic acid 8′-hydroxylase in stomata and vasculature to regulate local and systemic abscisic acid responses in *Arabidopsis*. Plant Physiol.

[CR42] Oliver MJ, Cushman JC, Koster KL (2010). Dehydration tolerance in plants. Methods Mol Biol.

[CR43] Pratt LH, Liang C, Shah M, Sun F, Wang H, Reid SP, Gingle AR, Paterson AH, Wing R, Dean R, Klein R, Nguyen HT, Ma HM, Zhao X, Morishige DT, Mullet JE, Cordonnier-Pratt MM (2005). Sorghum expressed sequence tags identify signature genes for drought, pathogenesis, and skotomorphogenesis from a milestone set of 16,801 unique transcripts. Plant Physiol.

[CR44] Sapeta H, Costa JM, Lourenço T, Maroco J, van der Linde P, Oliveira MM (2013). Drought stress response in *Jatropha curcas*: growth and physiology. Environ Exp Bot.

[CR45] Stern S, Dror T, Stolovicki E, Brenner N, Braun E (2007). Genome-wide transcriptional plasticity underlies cellular adaptation to novel challenge. Mol Syst Biol.

[CR46] Terzi R, Saruhan N, Saglam A, Nar H, Kadioglu A (2009). Photosystem II functionality and antioxidant system changes during leaf rolling in post-stress emerging *Ctenanthe setosa* exposed to drought. Acta Biol Hung.

[CR47] Tuteja N, Gill SS, Tuteja R (2011). Omics and plant abiotic stress tolerance.

[CR48] Upadhyaya H, Sahoo L, Panda SK (2013). Molecular physiology of osmotic stress in plants. Molecular stress physiology of plants.

[CR49] Wang D, Pan Y, Zhao X, Zhu L, Fu B, Li Z (2011). Genome-wide temporal–spatial gene expression profiling of drought responsiveness in rice. BMC Genomics.

[CR50] Wanke D, Kolukisaoglu HU (2010). An update on the ABCC transporter family in plants: many genes, many proteins, but how many functions?. Plant Biol.

[CR51] Wilkins O, Brautigam K, Campbell MM (2010). Time of day shapes *Arabidopsis* drought transcriptomes. Plant J.

[CR52] Xu C, Huang B (2010). Differential proteomic responses to water stress induced by PEG in two creeping bentgrass cultivars differing in stress tolerance. J Plant Physiol.

[CR53] Yan S, Tang Z, Su W, Sun W (2005). Proteomic analysis of salt stress-responsive proteins in rice root. Proteomics.

[CR54] Yang Y, He F, Yu L, Chen X, Lei J, Ji J (2007). Influence of drought on oxidative stress and flavonoid production in cell suspension culture of *Glycyrrhiza inflata Batal*. J Biosci (Z Naturforsch).

[CR55] Yang ZQ, Fan GQ, Cao YC, Wei ZZ, Liu F (2007). Establishment of the System of *in Vitro* Plantlet Regeneration of Different Autotetraploid *Paulownia* Plants. J Henan Agric Univ.

[CR56] Ye J, Fang L, Zheng H, Zhang Y, Chen J, Zhang Z, Wang J, Li S, Li R, Bolund L (2006). WEGO: a Web tool for plotting GO annotations. Nucleic Acids Res.

[CR57] Yoshimura K, Masuda A, Kuwano M, Yokota A, Akashi K (2008). Programmed proteome response for drought avoidance/tolerance in the root of a C(3) xerophyte (wild watermelon) under water deficits. Plant Cell Physiol.

[CR58] Zhang XS, Liu RN, Fan GQ, Zhao ZL, Deng MJ (2013). Study on the physiological response of tetraploid *Paulownia* to drought. J Henan Agric Univ.

[CR59] Zheng J, Fu J, Gou M, Huai J, Liu Y, Jian M, Huang Q, Guo X, Dong Z, Wang H, Wang G (2010). Genome-wide transcriptome analysis of two maize inbred lines under drought stress. Plant Mol Biol.

