# Supplementary figures and images for: Compatible solute, transporter protein, transcription factor, and hormone-related gene expression provides an indicator of drought stress in Paulownia fortunei
Source: Funct Integr Genomics. 2014 May 7;14(3):479–91. doi: 10.1007/s10142-014-0373-4 (PMC4137158; doi:10.1007/s10142-014-0373-4)

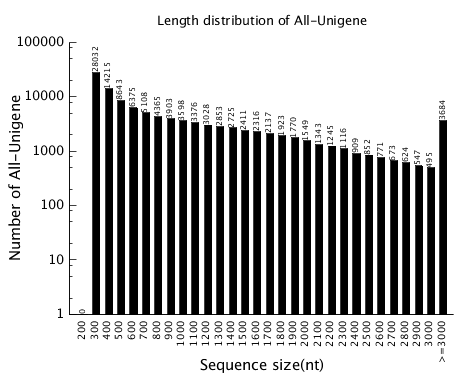

Supplement: Supplementary file 1 — (DOCX 24 kb) [file 10142_2014_373_MOESM1_ESM.docx]

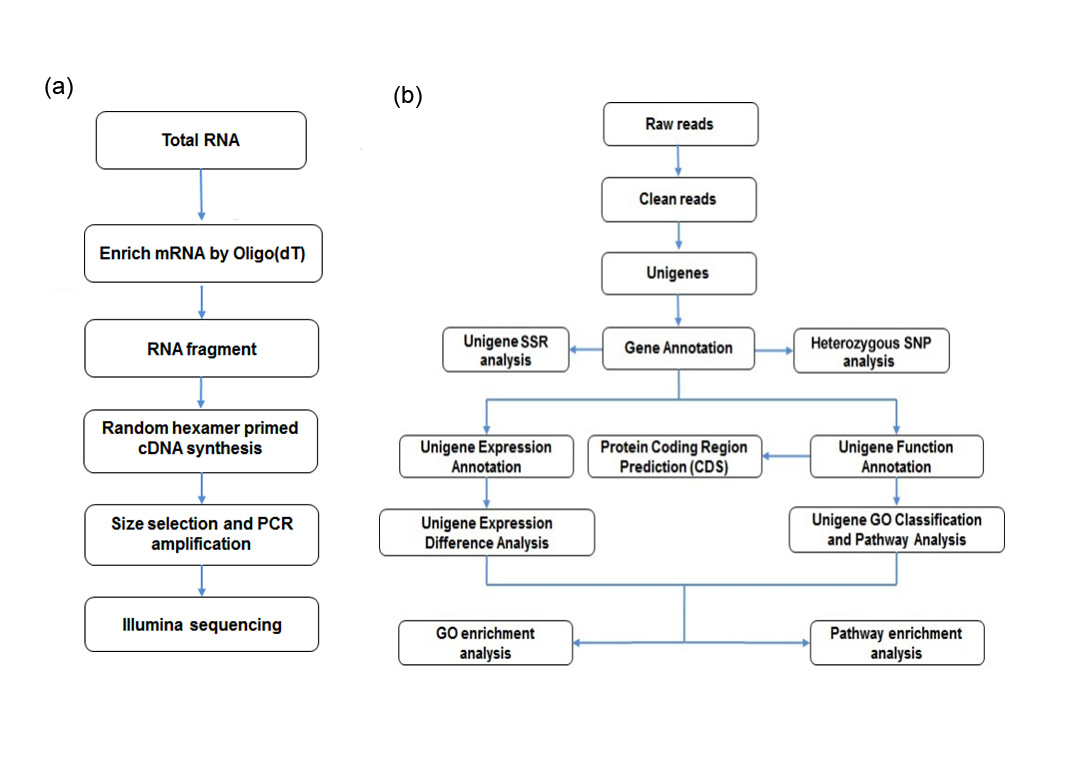

Supplement: Supplementary file 2 — (DOCX 162 kb) [file 10142_2014_373_MOESM2_ESM.docx]

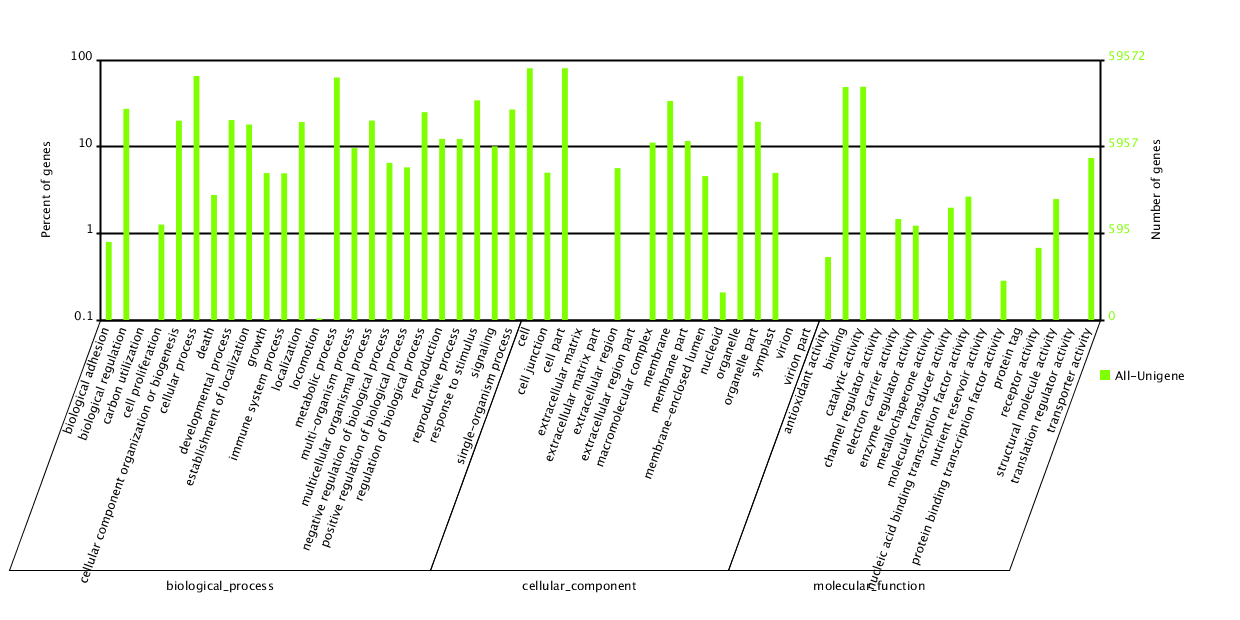

Supplement: Supplementary file 3 — (DOCX 120 kb) [file 10142_2014_373_MOESM3_ESM.docx]

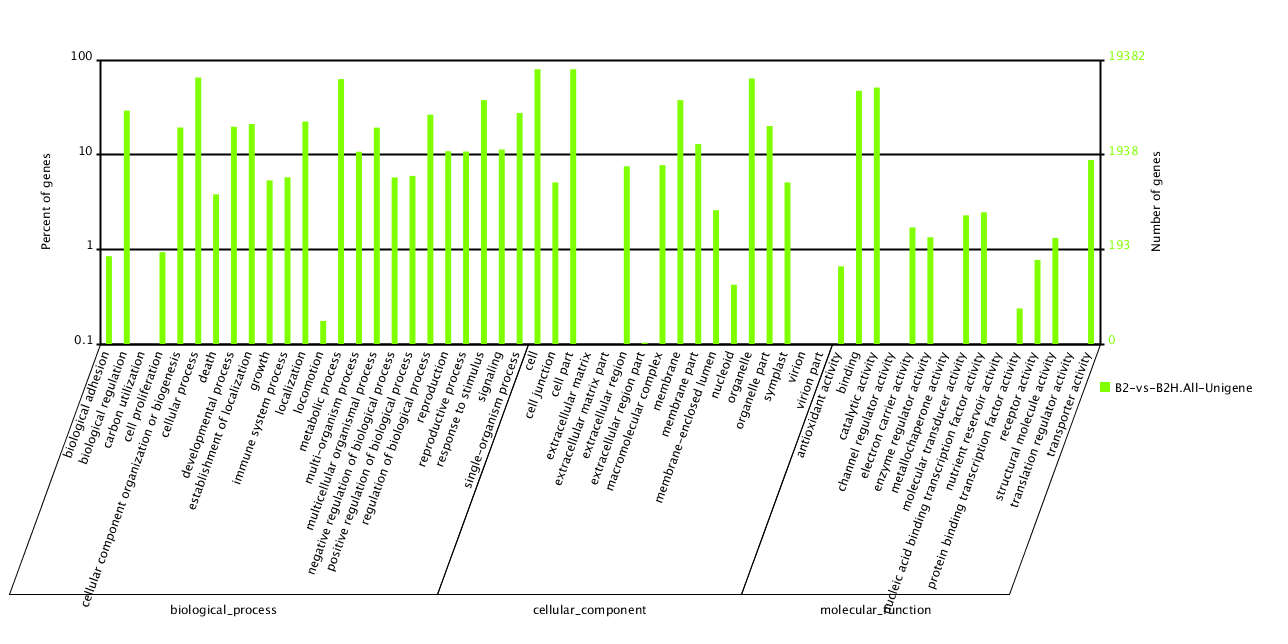

Supplement: Supplementary file 4 — (DOCX 115 kb) [file 10142_2014_373_MOESM4_ESM.docx]

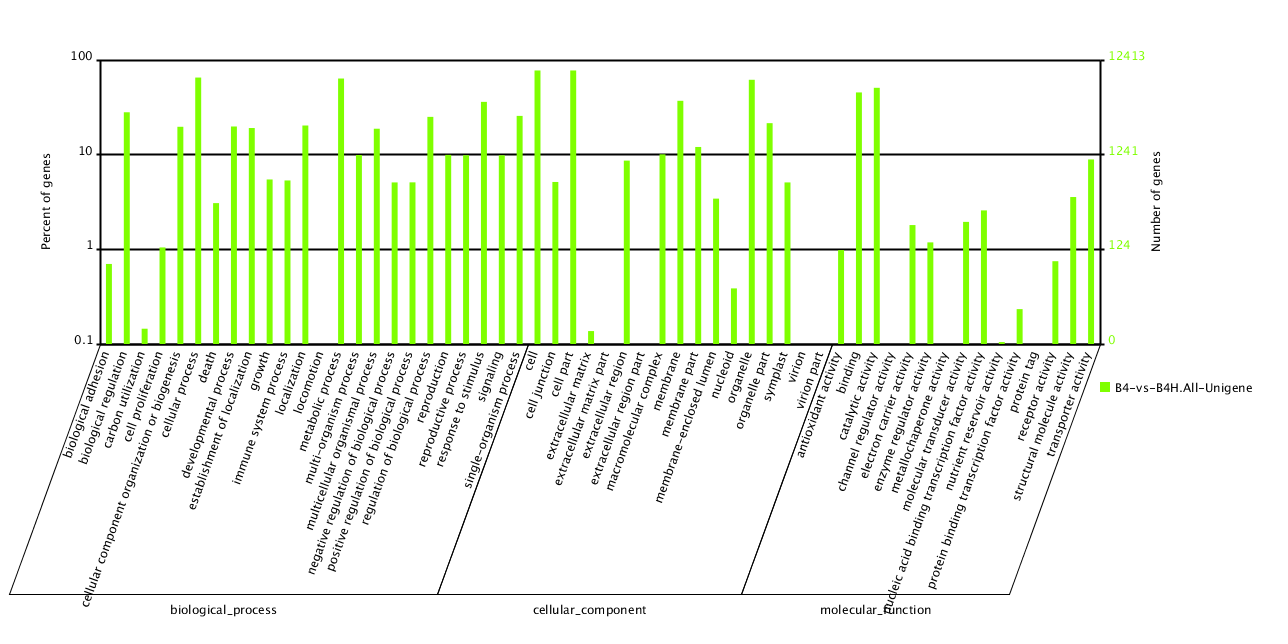

Supplement: Supplementary file 5 — (DOCX 115 kb) [file 10142_2014_373_MOESM5_ESM.docx]
